# The conjunctival transcriptome in Ethiopians after trichiasis surgery: associations with the development of eyelid contour abnormalities and the effect of oral doxycycline treatment

**DOI:** 10.12688/wellcomeopenres.15419.1

**Published:** 2019-09-04

**Authors:** Tamsyn Derrick, Esmael Habtamu, Zerihun Tadesse, E. Kelly Callahan, Abebaw Worku, Bizuayehu Gashaw, David Macleod, David C.W. Mabey, Martin J. Holland, Matthew J. Burton

**Affiliations:** 1 London School of Hygiene and Tropical Medicine, London, UK; 2 Kilimanjaro Christian Medical Centre, Moshi, Tanzania; 3 The Carter Center, Addis Ababa, Ethiopia; 4 The Carter Center, Atlanta, USA; 5 Amhara Regional Health Bureau, Bahirdar, Ethiopia

**Keywords:** Trichiasis, conjunctiva, eyelid contour abnormalities, post-operative, scarring, inflammation, doxycycline

## Abstract

**Background:** Surgery to correct trichiasis is a key component of the World Health Organisation trachoma control strategy, however unfavourable outcomes such as eyelid contour abnormalities (ECA) following surgery are relatively common. This study aimed to understand the transcriptional changes associated with the early development of ECA and the impact of doxycycline, which has anti-inflammatory and anti-fibrotic properties, upon these transcription patterns.

**Methods: **One thousand Ethiopians undergoing trichiasis surgery were enrolled in a randomised controlled trial following informed consent. Equal groups of randomly assigned individuals were orally administered with 100mg/day of doxycycline (n=499) or placebo (n=501) for 28 days. Conjunctival swabs were collected immediately prior to surgery and at one- and six-months post-surgery. 3’ mRNA sequencing was performed on paired baseline and one-month samples from 48 individuals; 12 in each treatment/ECA outcome group. qPCR validation was then performed for 46 genes of interest in 145 individuals who developed ECA at one month and 145 matched controls, using samples from baseline, one and six months.

**Results: **All treatment/outcome groups upregulated genes associated with wound healing pathways at one month relative to baseline, however no individual differences were detected between groups. The summed expression of a highly coexpressed cluster of pro-fibrotic genes was higher in patients that developed ECA in the placebo group relative to controls. qPCR validation revealed that all genes in this cluster and a number of other pro-inflammatory genes were strongly associated with ECA, however these associations were not modulated by trial arm.

**Conclusions:** The development of post-operative ECA is associated with overexpression of pro-inflammatory and pro-fibrotic genes including growth factors, matrix metalloproteinases, collagens and extracellular matrix proteins. There was no evidence that doxycycline modulated the association between gene expression and ECA.

## Introduction

Trachoma is a neglected tropical disease and the world’s leading infectious cause of blindness. Disease is initiated during childhood by repeated infection of the conjunctival epithelium with the intracellular bacterium *Chlamydia trachomatis*. This can lead to recurrent episodes of pathological inflammation which persist throughout the lives of some individuals and are a major risk factor for the development of scarring ^1^. Scarring of the tarsal conjunctiva causes the lid margin and eyelashes to turn inwards, known as entropion and trichiasis, respectively, such that the lashes brush against the cornea causing mechanical damage, pain and ultimately blindness. In 2017 over 200,000 people worldwide were managed for trichiasis; the disease is found predominantly in sub-Saharan Africa ^2^. Trachomatous inflammation and scarring can progress in the absence of detectable chlamydial infection ^1, 3^, therefore it is expected that trichiasis surveillance and management provisions will be required for many years in formerly endemic districts.

Trachoma is controlled at the population level through implementation of the SAFE Strategy: **S**urgery to correct trichiasis, **A**ntibiotics for infection control, and **F**acial cleanliness and **E**nvironmental improvements to prevent infection transmission. However, recurrence rates following trichiasis surgery range between 12–22% within 12 months and unfavourable outcomes such as the development of eyelid contour abnormalities (ECA) following surgery are relatively common, occurring in around 10% of patients ^4, 5^.

ECA involves notching or distortion of the eyelid margin. When ECA is severe the eyelid may no longer cover the globe of the eye when closed (lagophthalmos), increasing the risk of corneal damage and vision loss ^6^. Unfavourable aesthetic outcomes such as ECA may also dissuade patient’s friends or relatives from undergoing trichiasis surgery and may be stigmatising ^7– 9^. The pathophysiology of ECA is unknown, although it is likely due to an aberrant post-operative wound healing response involving excessive fibrosis and contraction of the tarsal conjunctiva. Previous studies have shown that a number of innate epithelial pro-inflammatory mediators ( *IL1B, S100A7, CXCL5*), matrix factors ( *SPARCL1, CTGF*, collagens) and matrix metalloproteinases (MMPs 7, 9, 12) are associated with progressive trachomatous scarring, trichiasis and recurrence post-surgery ^1, 10, 11^. MMPs are thought to facilitate fibrosis through remodelling the extracellular matrix (ECM) and enabling inflammatory cell infiltration. Failure of glaucoma filtration surgery is associated with excessive ECM deposition and contraction of subconjunctival tissue ^12^, and a number of collagen genes were found to be upregulated in the conjunctivae of patients with fibrotic outcomes following glaucoma surgery ^13^. These pro-inflammatory and matrix factors may also have a role in the development of ECA.

Doxycycline is a tetracycline-based antibiotic that is used clinically to treat sexually transmitted *C. trachomatis* infection. Several reports have presented evidence that doxycycline reduces inflammatory gene expression ^14– 17^ and it inhibits MMPs by reducing their expression, blocking their activation and inhibiting protease activity through directly binding zinc and calcium ions in the catalytic site ^18^. Doxycycline is used clinically to reduce inflammation and prevent fibrosis in diseases including rosacea, idiopathic pulmonary fibrosis and corneal erosion ^19– 21^. Li and colleagues presented data showing that doxycycline inhibited matrix remodelling and contraction and reduced MMP expression in primary fibroblasts isolated from individuals undergoing trichiasis surgery ^22^. These data therefore suggest that doxycycline could have a protective effect against the development of recurrent trichiasis and ECA. 

In order to address this question a randomised, double-blind, placebo-controlled trial was conducted to determine the impact of doxycycline treatment on post-operative trichiasis. One thousand Ethiopians eligible for trichiasis surgery were enrolled in the trial and the findings have been published in full elsewhere ^5^. Surprisingly, the cumulative proportion of individuals who developed recurrent trichiasis by 12 months after surgery was the same in both groups; 58/498 (12%) in the doxycycline group and 62/501 (12%) in the placebo group. There was also no difference in the proportion of individuals with mild, moderate or severe ECA at one, six or twelve months.

This study sought to understand the transcriptional changes associated with the early development of ECA following trichiasis surgery and the impact of doxycycline upon these transcription patterns. It was nested within the overall clinical trial and took place in parallel.

## Methods

### Ethics

This study was approved by the ethics committee of the London School of Hygiene & Tropical Medicine, Emory University Institutional Review Board, the Ethiopian National Health Research Ethics Review Committee and the Ethiopia Food, Medicine and Healthcare Administration and Controls Authority. The study was conducted in accordance with the Declaration of Helsinki and the International Conference on Harmonisation–Good Clinical Practice guidelines. Written informed consent or a witnessed thumbprint was requested from all study participants following detailed explanation of the study in Amharic.

### Surgery and examination

One thousand Ethiopians eligible for trichiasis surgery were enrolled in a randomised, double blind, placebo-controlled trial (registered with the Pan African Clinical Trials Registry, trial number PACTR201512001370307, registration date 29 ^th^ November 2015, registration URL: https://pactr.samrc.ac.za/TrialDisplay.aspx?TrialID=1370). Eligible individuals had evidence of trichiasis (one or more lashes touching the eye or evidence of epilation) and trachomatous scarring of the tarsal conjunctiva, were aged ≥ 18 years and had not had previous trichiasis surgery. Trichiasis surgery was performed using the posterior lamellar tarsal rotation (PLTR) procedure by experienced nurse-surgeons who had recently undergone refresher training. All study participants received 1% tetracycline eye ointment for self-administration twice a day for two weeks post-surgery. Participants were randomly assigned (1:1) to receive either 100mg/day doxycycline or 100mg/day placebo, orally administered, for 28 consecutive days after surgery.

Study participants were examined at baseline (immediately prior to surgery), 10 days, one month, six months and twelve months post-surgery. Examinations were performed by two standardised examiners with very strong agreement on primary outcome (k=0.92) (at baseline and twelve months (EH) and at one and 6 months), using 2.5X loupes and a torch. Clinical signs of trachoma (follicles, papillae and cicatrice; FPC) were graded using the detailed WHO grading system ^23^. Clinical signs or evidence of trichiasis, epilation and corneal scarring were recorded as previously described ^5^. At baseline, scarring was graded using the detailed FPC grading system, whereas after surgery scarring was graded using the post-operative scarring grading system ^24^. Sutures were removed on day 10. The primary outcome of the overall trial was the cumulative proportion of individuals who developed post-operative trichiasis by 12 months. Post-operative trichiasis was defined as minor (1–5 eyelashes touching the eye or evidence of epilation in less than a third of the eyelid margin) or major (≥6 eyelashes touching the eye or evidence of epilation in a third or more of the eyelid margin). ECA at one, six or 12 months was included as a secondary trial outcome. ECA was graded as previously described ^4, 6^: mild (vertical deviation from the natural contour less than 1 mm in height and affecting more than one third of horizontal eyelid length); moderate (vertical deviation from the natural contour 1–2 mm in height or affecting one third to two thirds of horizontal eyelid length) and severe (vertical deviation from the natural contour more than 2 mm in height or a defect more than two thirds the horizontal eyelid length). Mild ECA is considered clinically non-significant whereas moderate and severe ECA are considered clinically significant. Drug adherence was monitored through interviews and by counting the number of capsules remaining. Full trial details and results are published elsewhere ^5^.

### Sample collection

Swabs from the tarsal conjunctiva were collected at baseline and at one, six- and twelve-months post-surgery. A sterile polyester swab (Puritan) was passed across the conjunctival surface four times, with a quarter-turn on each pass. Swabs were stored in RNAlater (Thermo Fisher Scientific) at 4°C overnight and then long-term at -80°C. Swabs were shipped (on dry ice) to Kilimanjaro Christian Medical Centre (KCMC), Tanzania for processing. RNA and DNA were extracted from swabs using Norgen RNA/DNA purification kit (Norgen Biotek) following manufacturer’s instructions.

### Chlamydia trachomatis testing

DNA samples were tested for *C. trachomatis* using a published qPCR assay ^25^. Samples were tested in duplicate using the triplex assay which detects a human endogenous control gene ( *RPP30*), *C. trachomatis* plasmid (pORF2) and chromosomal ( *omcB*) targets. Each reaction of 20μl contained 10μl TaqMan Multiplex Master Mix (Thermo Fisher Scientific), 300nM of each primer and probe (Thermo Fisher Scientific), 4μl water and 4μl sample DNA. Samples were tested on a ViiA7 thermal cycler (Thermo Fisher Scientific) with the following conditions: a 20 second hold at 95°C, followed by 40 cycles of 95°C for one second and 60°C for 20 seconds. Samples were determined *C. trachomatis* positive if *RPP30* and both chlamydial targets (pORF2 and *omcB*) were positive in either or both replicates. Target concentration was calculated by extrapolating from a standard curve.

### 3’RNA sequencing

3’RNA sequencing was performed on paired baseline and one-month samples from 48 individuals; 12 in each treatment/outcome group (Placebo-Good outcome, Placebo-Poor outcome, Doxycycline-Good outcome, Doxycycline-Poor outcome). All 48 individuals had moderate trachomatous scarring and no eyelid distortion at baseline (Detailed FPC scarring grade 2). Individuals with Poor outcomes had scarring distortion (SC4 or SC5) at one and six months, grade 2 or 3 ECA at one month, and grade 1, 2 or 3 ECA at six months. Individuals with Good outcomes had no scarring distortion or ECA at one and six months. Potential participants were selected at random (randomisation was performed in STATA v15) from each of the four treatment/outcome groups.

Library preparation was performed using QuantSeq 3’ mRNA-Seq Library Prep Kit FWD for Illumina (Lexogen GmbH, Austria), following the manufacturer’s instructions. 3’ RNA sequencing generates one read per transcript at the 3’ end of polyadenylated mRNA and maintains strand-specificity. Sample RNA concentration was measured using Qubit HS RNA kits (Thermo Fisher Scientific) and RNA quality was tested using Agilent RNA 6000 Pico chips (Agilent Technologies, USA). Sample concentrations were normalised to 10ng/μl and 5μl RNA was used for library preparation. The PCR Add-on kit (Lexogen GmbH) was used during sample preparation to generate libraries that were not under or over-cycled. Final library concentration was tested using Agilent DNA chips and libraries were normalised to 6nM prior to pooling. Libraries were shipped to Lexogen GmbH for quality control and sequencing on two lanes of an Illumina HiSeq (Illumina).

Bam files were converted to FastQ using Picard and FastQ files were uploaded to BlueBee Genomics Platform (BlueBee). FastQ files were trimmed and quality filtered using the default Integrated QuantSeq FWD workflow. In brief, Bbduk was used to trim low-quality tails, adapter contamination and polyA read-through, reads were aligned to human genome version GRCh38.77 using STAR Aligner and reads were counted using HTSeq-count with kit-specific options. Raw and processed RNAseq data are deposited within the NCBI GEO public database (accession number GSE135455).

### Gene expression

Of the 1000 participants in the clinical trial, a total of 145 had developed moderate or severe ECA (grade 2 or 3) at the one-month time-point ^5^. These 145 individuals and 145 age, sex and trial arm matched individuals without ECA (none or mild ECA (grade 0 or 1)) at one month were selected for qPCR validation of gene expression, using samples from baseline, one month and six months; making a total of 870 samples.

RNA was converted to cDNA using SuperScript VILO cDNA synthesis kits (Thermo Fisher Scientific) following the manufacturer’s instructions. The relative abundance of 46 genes of interest and endogenous control genes HPRT1 and GAPDH were quantified in each sample by qPCR using TaqMan Microfluidic 384-well Array Cards (Thermo Fisher Scientific). qPCR was performed using TaqMan Universal Master Mix (Thermo Fisher Scientific) on a ViiA7 thermal cycler following the manufacturer’s instructions. Genes of interest were selected based on 3’RNA sequencing results, those hypothesised to be involved in the pathogenesis of scarring trachoma and genes reported to be immunomodulated by doxycycline ^1, 10, 14– 17, 22, 26, 27^.

After running the first 239 samples and noting poor amplification or failure of a number of samples on TaqMan Array Cards, all remaining samples were tested for *HPRT1* expression by single-plex qPCR for quality screening *.* qPCR was performed using a HPRT1 TaqMan Assay (the same as that used on the Array Card (assay ID: Hs02800695_m1; Thermo Fisher Scientific) and TaqMan Universal Master Mix on a ViiA7 thermal cycler following the manufacturer’s instructions. A cycle threshold value of 31 was determined as the screening cut-off based on data from Array Cards that had already been run. Of the 631 remaining samples that had not yet been run on Array Cards, 63 had *HPRT1* qPCR CT values >31. After excluding failed samples and those that did not pass *HPRT1* screening, 772/870 samples remained with gene expression data.

### Data analysis

***3’RNA sequencing analysis.*** Read count data were downloaded from BlueBee and imported to R ( www.R-project.org, R version 3.5.1) for analysis. Read count data were analysed for differential expression using DEseq2, adjusting for sex and age ^28^. Genes were retained in the analysis that had a read count of 10 or more in 48 or more samples (n=11424). The DEseq2 package internally corrects for library size and a plot of the Cook’s distance between samples revealed no sample outliers (data not shown) therefore no samples were excluded from the analyses. Samples were assigned to four groups; doxycycline and good outcome, doxycycline and ECA, placebo and good outcome, placebo and ECA. The differences in gene expression at one month relative to baseline in paired samples from each group was compared initially. The differences in gene expression between baseline and one month within each group were then contrasted between groups in pairwise comparisons. P values were adjusted by the Benjamini and Hochberg method within the DEseq2 package. Principal component analysis was performed within DEseq2 using read count data. The matrix of raw read counts for all 96 samples and 11424 genes was entered into Miru v1.0 ( https://kajeka.com; now called Graphia) to investigate clusters of samples with highly correlated expression values. The Pearson correlation cutoff was increased to the maximum possible whilst still retaining all 96 samples (cutoff = 0.93).

Genes with an adjusted P value (Padj) <0.1 and FC>1.5 within each group at one month relative to baseline were entered into Gene Set Over-representation analysis in Consensus Path Database (CPDB, http://consensuspathdb.org), using all 11424 genes as background, minimum overlap with input list=2 and P value cutoff=0.01.

The matrix of raw read counts for all 96 samples and 11424 genes was transposed and entered again into Miru to investigate networks of co-expressed genes. Genes were clustered by Pearson correlation coefficient using a cutoff >=0.88. The genes in each cluster were also entered into Gene Set Over-representation analysis in CPDB using 11424 genes as background. For each individual, the read counts for all genes in each cluster were summed, creating a single summative value per cluster. These summative values were then contrasted between one-month post-surgery treatment/outcome groups using linear regression, using the placebo/good outcome group as the reference group and adjusting for baseline (pre-surgery) expression, age and sex.

***qPCR.*** Gene expression results were imported into STATA v15 for analysis. Cycle threshold values in each sample were normalised to the endogenous control gene (HPRT1) to generate delta CT values ^29^. For quality control purposes, both genes and observations (a sample from a participant at one time-point) with 10% missing data were excluded. This resulted in the exclusion of two genes, *PAPPA* and *TRGV9*, and 22/772 observations, leaving 46 genes (44 genes of interest in addition to *HPRT1* and *GAPDH*) and 751 observations in the analysis.

A binary ECA variable was created for analysis purposes based on the clinical significance criteria at each of the one- and six-month time-points: none or mild ECA (grades 0 or 1) = 0 and moderate and severe ECA (grades 2 or 3) = 1. In order to determine whether any of the shortlisted genes were associated with ECA, mixed effects logistic regression models were constructed with binary ECA as the dependant variable and the delta CT values of each gene in turn as the independent variable, adjusting for trial arm, age and sex, and using data from baseline, one and six months. Participant identification number was included as a random effect to account for samples from the same individuals being used from multiple timepoints. *C. trachomatis* was not adjusted for in these analyses due to the very low number of infections detected. Following these initial models, the models were performed again including an interaction term between gene expression and treatment arm, to determine whether treatment arm modified the effect of gene expression on ECA outcome. A likelihood ratio test was then performed to determine whether the model including the interaction explained the data better than the model without the interaction. The Benjamini and Hochberg method was used to control for a false discovery rate of 5% ^30^.

## Results

### RNA sequencing

3’RNA sequencing was performed on samples from 48 individuals at baseline (immediately prior to surgery) and at one-month post-surgery, making a total of 96 samples. All 48 individuals had moderate trachomatous scarring and no eyelid distortion at baseline (FPC scarring grade 2). Of these 48 individuals, there were 12 in each treatment/outcome group: Placebo-Good outcome, Placebo-Poor outcome, Doxycycline-Good outcome, Doxycycline-Poor outcome. The demographic and clinical characteristics of these 48 individuals are shown in Table 1.

**Table 1.** Clinical and demographic details of 48 participants included in the RNAsequencing study.

	Placebo-Good	Placebo-Poor	Doxycycline-Good	Doxycycline-Poor
Total	12	12	12	12
Age (mean, range)	40.3 (20-60)	56.5 (35-80)	50.7 (30-75)	56.8 (41-75)
Sex (female/12)	9	10	8	8
*C. trachomatis* detected	0	0	0	0
ECA at 1 month: none	12	0	12	0
ECA at 1 month: 2	0	8	0	11
ECA at 1 month: 3	0	4	0	1
Scarring at 1 month: SC3	12	0	12	0
Scarring at 1 month: SC4	0	10	0	11
Scarring at 1 month: SC5	0	2	0	1

After filtering out genes that had a read count <10 in 48 or more samples, 11,424 genes were retained. RNA sequencing analysis revealed that within each of the four groups (Placebo-Good; Placebo-Poor; Doxycycline-Good; Doxycycline-Poor), a large number of genes were differentially regulated at one month relative to baseline ( Table 2). In each group, roughly twice as many genes were upregulated as were downregulated. More genes were differentially regulated in the Poor groups relative to the Good groups in both placebo and doxycycline treatment arms. Whilst the number of differentially expressed genes was similar in both Poor groups, there were a higher number of differentially expressed genes in the doxycycline-Good group relative to the placebo-Good group.

**Table 2.** The number of differentially expressed genes within each treatment/outcome group, at one-month relative to baseline.

Contrast	Genes Padj <0.1	Upregulated (FC>1.5)	Downregulated (FC<0.67)
Placebo-Good outcome (1m vs. baseline) ^a^	395	221	101
Placebo-Poor outcome (1m vs. baseline) ^a^	1659	788	395
Doxycycline-Good outcome (1m vs. baseline) ^a^	993	512	216
Doxycycline-Poor outcome (1m vs. baseline) ^a^	1497	651	433

^a^ Upregulated genes had higher expression at 1 month relative to baseline

Differentially expressed genes (Padj<0.1, FC>1.5) within each treatment/outcome group at one month relative to baseline were entered into pathway analysis. The ten most enriched pathways for each group are shown in Table 3. All pathways were reflective of wound healing responses, including ECM organisation, collagen formation and interactions between cells and the ECM. The two Poor outcome groups were enriched for several immune response pathways that were not present in the top ten enriched pathways of the Good outcome groups ( Table 3).

**Table 3.** The ten most enriched pathways within each treatment/outcome group at one-month post-surgery relative to baseline. Pathways are ranked in order of significance. For all pathways shown, the enrichment P value was <2 x 10 ^-10^.

Placebo-Good	Placebo-Poor	Doxycycline-Good	Doxycycline-Poor
Extracellular matrix organization	Extracellular matrix organization	Extracellular matrix organization	Extracellular matrix organization
Collagen formation	Beta1 integrin cell surface interactions	Collagen formation	Collagen formation
Beta1 integrin cell surface interactions	Collagen formation	Beta1 integrin cell surface interactions	Collagen biosynthesis and modifying enzymes
Collagen chain trimerization	Cell adhesion molecules (CAMs) - Homo sapiens (human)	Integrin	Class A/1 (Rhodopsin-like receptors)
Collagen biosynthesis and modifying enzymes	Hematopoietic cell lineage - Homo sapiens (human)	Collagen biosynthesis and modifying enzymes	Peptide ligand-binding receptors
ECM-receptor interaction - Homo sapiens (human)	Staphylococcus aureus infection - Homo sapiens (human)	ECM-receptor interaction - Homo sapiens (human)	Beta1 integrin cell surface interactions
Degradation of the extracellular matrix	Collagen chain trimerization	Cell adhesion molecules (CAMs) - Homo sapiens (human)	Collagen chain trimerization
Integrin	Complement and coagulation cascades - Homo sapiens (human)	ECM proteoglycans	Interleukin-4 and 13 signalling
Focal Adhesion	Collagen biosynthesis and modifying enzymes	Beta3 integrin cell surface interactions	Regulation of Complement cascade
Focal adhesion - Homo sapiens (human)	Classical antibody-mediated complement activation	Degradation of the extracellular matrix	ECM proteoglycans

The differences in gene expression at one month relative to baseline within each treatment/outcome group were then contrasted between groups. Pairwise comparisons revealed almost no significant differences in gene expression between groups ( Table 4). Four genes (PSD3, ZEB1, RAD17, RNF181) were differentially expressed in the placebo group, and one (NUAK2) in the doxycycline group, between Good and Poor outcomes (Padj<0.1, FC>1.5). There were no overall differences between placebo and doxycycline groups (irrespective of outcome). Four genes were differentially expressed between Good and Poor outcomes (irrespective of treatment group); two known genes (PTP4A1, FBXO31) and two uncharacterised genes (ENSG00000256940, ENSG00000257742).

**Table 4.** Pairwise contrasts of within-group differences (between baseline and one-month samples) between treatment/outcome groups.

Contrast	Genes Padj <0.1	Upregulated (FC>1.5)	Downregulated (FC<0.67)
Placebo-Poor vs. Placebo-Good ^b^	4	3	1
Doxycycline-Poor vs. Doxycycline-Good ^b^	1	-	1
Placebo-Good vs. Doxycycline-Good	0	-	-
Placebo-Poor vs. Doxycycline-Poor	0	-	-
All placebo vs. Doxycycline	0	-	-
All Good vs. Poor outcome	4	3	1

^b^ Upregulated genes had higher expression in the Poor outcome group

**Figure 1.**
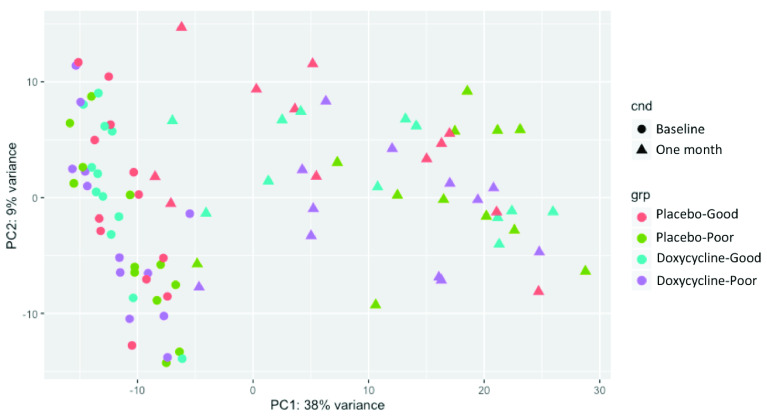
Principal component analysis of read count data from baseline and one-month samples.

Principal component analysis of read count data revealed considerable within-group variation in the one-month samples ( Figure 1). The raw read count matrix for all 96 samples and 11424 genes was entered into coexpression analysis using a Pearson correlation cut-off of 0.93. This generated three clusters of samples (data not shown). Nine samples were not included in any cluster and had no class. Treatment/outcome group samples at baseline and one month appeared to distribute evenly across all three clusters, with the exception of the placebo-Poor one-month samples, which were all found in cluster one (with the exception of one sample that had no class).

The raw read count matrix for all samples was inverted and analysed again for networks of co-expressed genes. Using a Pearson correlation cut-off of 0.88, 40 clusters of genes were identified. Genes in each of the ten largest clusters (containing ≥9 genes) were entered into pathway analysis using the background of 11424 genes included in differential expression testing. The largest cluster, cluster 1, was highly enriched for pathways associated with wound healing ( *Extended data*). With the exception of cluster 8, which was enriched for phagocytosis-related genes, all other clusters were enriched for cell cycle and pathways of general cellular processes ( *Extended data*). In order to determine whether these tightly co-expressed gene clusters were differentially expressed between treatment/outcome groups, the read counts for all genes in each cluster were summed for each individual. The expression of each cluster-sum was then compared between groups at one month by linear regression, adjusting for cluster-sum expression at baseline (prior to surgery), age and sex. 

The summative expression of the 20 genes in cluster one, which was strongly enriched for pro-fibrotic pathways, was higher in those with a Poor outcome relative to those with a Good outcome in the placebo group (P=0.017). The summative expression of cluster one genes was not different in the doxycycline group in those with Good or Poor outcomes relative to the placebo-Good group however. There was no evidence of differential expression of clusters 2–10 between treatment/outcome groups.

### Gene expression

The genes in cluster 1, which were enriched for pro-fibrotic pathways and were more highly expressed in individuals who developed a Poor outcome relative to Good outcome in the placebo arm, were tested by qPCR in a larger sample of individuals to validate their differential expression. A number of other genes hypothesised to be involved in the development of ECA or to be immunomodulated by doxycycline were also tested ^1, 10, 14– 17, 22, 26, 27^. Of the 1000 participants in the clinical trial, a total of 145 had developed moderate or severe ECA at one month ^5^. These 145 individuals and 145 age, sex and trial arm matched individuals who had not developed a Poor outcome by 1 month were selected for qPCR validation of gene expression, using samples from baseline, one month and six months ( Table 5).

**Table 5.** Clinical and demographic details of 290 participants used for qPCR validation.

Outcome	Good outcome		Poor outcome	
Treatment arm	Placebo	Doxycycline	Placebo	Doxycycline
Total	72	73	72	73
Age (mean, range)	54.5 (26–80)	56.9 (34–82)	54.9 (24–80)	57.5 (30–80)
Sex (female/2)	57	53	57	53
***C. trachomatis detected:***				
Baseline	3	0	0	1
1 month	1	0	0	0
6 months	2	0	0	1
***Scarring at baseline:***				
C0	0	0	0	0
C1	2	2	4	7
C2	59	61	61	57
C3	11	10	7	9
***ECA at one month:***				
none	51	55	0	0
Grade 1	21	18	0	0
Grade 2	0	0	64	60
Grade 3	0	0	8	13
***ECA at six months:***				
none	64	68	26	22
Grade 1	6	3	23	25
Grade 2	2	2	21	20
Grade 3	0	0	2	4

Of the 145 individuals with Poor outcomes (moderate or severe ECA) at one month, 48 had improved to ECA grade 0 at six months; 22 in the doxycycline and 26 in the placebo treatment arms. A binary ECA variable was therefore generated for qPCR analyses, defined as none/mild (ECA grade 0/1) and moderate/severe (ECA grade 0/1) at each of the one and six-month time-points. There was no difference in baseline trichiasis grade (P=0.8), baseline papillary inflammation (P=0.39), baseline conjunctival scarring grade (P=0.71), or surgeon (P=0.72) between those with and without ECA.

Two genes were filtered out prior to analysis due to having >10% missing data ( *PAPPA* and *TRGV9*), leaving 44 genes of interest in the analysis. Twenty-six of these genes were strongly associated with ECA (at the FDR adjusted P value threshold of 0.0036), all of which were upregulated, reflected by an odds ratio less than one (delta CT values were used in the analysis) ( Table 6).

**Table 6.** Genes associated with ECA, adjusting for trial arm, age and sex. Data from baseline, one- and six-months post-surgery were included. Negative odds ratios (OR) indicate that gene expression was higher in individuals with ECA. CI = confidence interval. P values <0.0036 are considered significant after adjustment for the 5% FDR.

	Gene expression			Trial Arm			Age			Sex		
Target	OR	95% CI	P value	OR	95% CI	P value	OR	95% CI	P value	OR	LCI - UCI)	P value
AEBP1 (AE Binding Protein 1)	0.63	(0.57 - 0.70)	**9.96E-20**	1.13	(0.78 - 1.63)	0.5299	1.02	(1.00 - 1.03)	0.0405	1.24	(0.80 - 1.91)	0.3321
CCL2 (chemokine (C-C motif) ligand 2)	0.40	(0.23 - 0.69)	**0.0009**	1.08	(0.54 - 2.16)	0.8183	1.03	(1.00 - 1.07)	0.0815	2.11	(0.84 - 5.33)	0.1144
CCL3 (chemokine (C-C motif) ligand 3)	0.65	(0.59 - 0.73)	**4.31E-16**	1.04	(0.72 - 1.49)	0.8403	1.01	(1.00 - 1.03)	0.1180	1.18	(0.77 - 1.81)	0.4439
CCL4 (chemokine (C-C motif) ligand 4)	0.55	(0.33 - 0.92)	0.0221	1.05	(0.60 - 1.86)	0.8554	1.02	(0.99 - 1.05)	0.1451	1.30	(0.65 - 2.63)	0.4587
CD247 (T-cell surface glycoprotein CD3 zeta chain)	0.71	(0.39 - 1.29)	0.2672	1.03	(0.64 - 1.67)	0.9023	1.02	(0.98 - 1.06)	0.2811	1.12	(0.62 - 2.01)	0.7131
CD68 (Cluster of Differentiation 68)	0.55	(0.20 - 1.47)	0.2305	1.03	(0.63 - 1.68)	0.9103	1.02	(0.98 - 1.05)	0.2903	1.11	(0.61 - 2.01)	0.7405
CHUK (conserved helix-loop-helix ubiquitous kinase)	0.60	(0.46 - 0.78)	**0.0001**	1.01	(0.72 - 1.43)	0.9429	1.01	(1.00 - 1.03)	0.1467	1.07	(0.72 - 1.61)	0.7291
COL1A1 (Collagen, type I, alpha 1)	0.78	(0.74 - 0.81)	**2.09E-28**	1.07	(0.73 - 1.56)	0.7382	1.01	(1.00 - 1.03)	0.0877	1.27	(0.81 - 1.99)	0.2989
COL3A1 (Collagen, type 3, alpha 1)	0.75	(0.71 - 0.79)	**5.05E-28**	1.07	(0.73 - 1.56)	0.7387	1.01	(1.00 - 1.03)	0.0724	1.34	(0.85 - 2.11)	0.2044
COL5A1 (Collagen, type 5, alpha 1)	0.71	(0.67 - 0.76)	**7.02E-27**	1.09	(0.74 - 1.60)	0.6608	1.02	(1.00 - 1.03)	0.0342	1.22	(0.78 - 1.92)	0.3837
COL6A1 (Collagen, type 6, alpha 1)	0.71	(0.67 - 0.75)	**4.67E-29**	1.10	(0.75 - 1.61)	0.6390	1.02	(1.00 - 1.03)	0.0371	1.30	(0.83 - 2.05)	0.2554
COL7A1 (Collagen, type 7, alpha 1)	0.72	(0.68 - 0.77)	**3.37E-26**	1.08	(0.74 - 1.58)	0.6877	1.02	(1.00 - 1.03)	0.0375	1.22	(0.77 - 1.91)	0.3942
CSF1R (Colony stimulating factor 1 receptor)	0.83	(0.54 - 1.27)	0.3927	1.05	(0.65 - 1.69)	0.8530	1.02	(0.98 - 1.05)	0.3481	1.13	(0.62 - 2.04)	0.6901
CTGF (Connective Tissue Growth Factor)	0.56	(0.51 - 0.62)	**2.75E-30**	1.08	(0.73 - 1.59)	0.7028	1.02	(1.00 - 1.04)	0.0136	1.35	(0.85 - 2.14)	0.2069
CXCL5 (C-X-C motif chemokine 5)	0.69	(0.63 - 0.75)	**3.59E-15**	0.99	(0.69 - 1.42)	0.9643	1.01	(0.99 - 1.02)	0.3647	1.00	(0.66 - 1.54)	0.9826
CXCL8 (C-X-C motif chemokine 8)	0.57	(0.24 - 1.35)	0.2005	1.00	(0.67 - 1.50)	0.9948	1.01	(0.99 - 1.04)	0.2857	0.98	(0.61 - 1.59)	0.9446
FERMT2 (Fermitin family homolog 2)	0.56	(0.49 - 0.64)	**2.13E-17**	1.04	(0.72 - 1.49)	0.8501	1.02	(1.00 - 1.03)	0.0210	1.31	(0.85 - 2.01)	0.2193
FKBP10 (FK506-binding protein 10)	0.61	(0.56 - 0.67)	**1.84E-27**	1.12	(0.76 - 1.65)	0.5540	1.02	(1.00 - 1.04)	0.0310	1.41	(0.90 - 2.22)	0.1377
FN1 (Fibronectin)	0.75	(0.72 - 0.79)	**4.23E-28**	1.07	(0.73 - 1.57)	0.7113	1.02	(1.00 - 1.04)	0.0253	1.25	(0.79 - 1.96)	0.3364
FUT4 (Fucosyltransferase 4 (alpha (1,3) fucosyltransferase, myeloid-specific))	0.67	(0.57 - 0.77)	**9.89E-08**	0.96	(0.67 - 1.36)	0.8059	1.01	(1.00 - 1.03)	0.0900	1.06	(0.70 - 1.60)	0.7823
IFNG (Interferon Gamma)	1.18	(0.84 - 1.65)	0.3502	1.03	(0.63 - 1.66)	0.9139	1.01	(0.98 - 1.04)	0.4366	1.04	(0.59 - 1.84)	0.8819
IKBKB (inhibitor of nuclear factor kappa-B kinase subunit beta)	0.28	(0.07 - 1.07)	0.0627	0.97	(0.57 - 1.66)	0.9111	1.02	(0.99 - 1.06)	0.1533	1.08	(0.58 - 2.02)	0.8114
IL10 (Interleukin 10)	0.52	(0.25 - 1.08)	0.0788	0.98	(0.57 - 1.66)	0.9316	1.02	(0.99 - 1.05)	0.1989	1.21	(0.63 - 2.32)	0.5771
IL17A (Interleukin 17 Alpha)	1.23	(0.96 - 1.58)	0.0974	1.06	(0.73 - 1.54)	0.7627	1.01	(0.99 - 1.03)	0.2777	1.22	(0.77 - 1.94)	0.3942
IL18 (Interleukin 18)	1.23	(1.00 - 1.53)	0.0520	1.03	(0.73 - 1.46)	0.8452	1.01	(1.00 - 1.03)	0.1265	1.10	(0.73 - 1.64)	0.6489
IL1B (Interleukin 1 Beta)	0.48	(0.30 - 0.79)	**0.0036**	1.06	(0.56 - 2.02)	0.8619	1.02	(0.99 - 1.05)	0.1537	1.12	(0.52 - 2.41)	0.7727
IL6 (Interleukin 6)	0.41	(0.26 - 0.66)	**0.0002**	0.98	(0.47 - 2.04)	0.9603	1.03	(1.00 - 1.07)	0.0751	1.22	(0.51 - 2.94)	0.6560
MMP12 (Matrix Metalloproteinase 12)	0.56	(0.27 - 1.15)	0.1154	1.09	(0.74 - 1.61)	0.6500	1.02	(0.99 - 1.04)	0.2045	0.98	(0.63 - 1.53)	0.9348
MMP1 (Matrix Metalloproteinase 1)	0.55	(0.49 - 0.62)	**1.81E-22**	1.07	(0.74 - 1.56)	0.7128	1.02	(1.00 - 1.03)	0.0415	1.26	(0.81 - 1.98)	0.3064
MMP7 (Matrix Metalloproteinase 7)	0.56	(0.35 - 0.91)	0.0184	0.92	(0.52 - 1.63)	0.7852	1.01	(0.99 - 1.04)	0.2882	1.11	(0.57 - 2.16)	0.7698
MMP9 (Matrix Metalloproteinase 9)	0.37	(0.21 - 0.66)	**0.0007**	0.99	(0.49 - 1.99)	0.9821	1.03	(0.99 - 1.06)	0.0991	1.43	(0.60 - 3.37)	0.4180
MS4A1 (B-lymphocyte antigen CD20)	0.85	(0.67 - 1.07)	0.1686	1.02	(0.62 - 1.68)	0.9461	1.02	(0.99 - 1.05)	0.2175	1.00	(0.55 - 1.80)	0.9923
NCAM1 (Neural cell adhesion molecule (NCAM))	0.80	(0.66 - 0.96)	0.0180	1.04	(0.74 - 1.47)	0.8043	1.01	(1.00 - 1.03)	0.0679	1.07	(0.71 - 1.60)	0.7447
NFKB1 (Nuclear factor NF-kappa-B p105 subunit)	0.30	(0.05 - 1.88)	0.1963	0.97	(0.63 - 1.48)	0.8751	1.02	(0.99 - 1.05)	0.2678	1.09	(0.65 - 1.81)	0.7494
PRRX1 (Paired related homeobox 1)	0.66	(0.61 - 0.71)	**2.99E-25**	1.08	(0.73 - 1.58)	0.7114	1.01	(1.00 - 1.03)	0.1690	1.25	(0.79 - 1.98)	0.3330
PTPRC (Protein tyrosine phosphatase, receptor type, C)	0.58	(0.48 - 0.70)	**1.43E-08**	1.03	(0.73 - 1.47)	0.8530	1.02	(1.00 - 1.03)	0.0206	1.02	(0.68 - 1.54)	0.9189
S100A7 (S100 calcium-binding protein A7)	1.01	(0.92 - 1.10)	0.8650	1.04	(0.65 - 1.68)	0.8562	1.02	(0.98 - 1.06)	0.4012	1.14	(0.62 - 2.07)	0.6772
SPARC (secreted protein acidic and rich in cysteine)	1.11	(0.93 - 1.33)	0.2403	1.06	(0.64 - 1.76)	0.8174	1.01	(0.99 - 1.04)	0.2924	1.06	(0.59 - 1.91)	0.8470
SPARCL1 (SPARC-like protein 1)	0.67	(0.63 - 0.72)	**5.82E-27**	1.05	(0.72 - 1.53)	0.8048	1.02	(1.00 - 1.03)	0.0543	1.31	(0.84 - 2.06)	0.2308
TGFB1I1 (Transforming growth factor beta-1-induced transcript 1 protein)	0.57	(0.51 - 0.64)	**1.70E-21**	1.06	(0.73 - 1.53)	0.7591	1.02	(1.00 - 1.03)	0.0235	1.35	(0.87 - 2.09)	0.1823
TGFBI (Transforming growth factor, beta-induced, 68kDa)	0.35	(0.28 - 0.44)	**4.07E-19**	0.97	(0.67 - 1.40)	0.8827	1.03	(1.01 - 1.05)	**0.0010**	1.33	(0.86 - 2.06)	0.2046
TNF (Tumor Necrosis Factor)	0.71	(0.44 - 1.15)	0.1640	1.01	(0.61 - 1.66)	0.9771	1.02	(0.99 - 1.05)	0.2054	1.19	(0.64 - 2.20)	0.5900
TPM2 (β-Tropomyosin)	0.60	(0.54 - 0.65)	**1.16E-28**	1.09	(0.74 - 1.60)	0.6589	1.02	(1.00 - 1.03)	0.0527	1.18	(0.75 - 1.86)	0.4838
VCAN (Versican)	0.62	(0.57 - 0.67	**2.70E-28**	1.05	(0.71 - 1.54	0.8183	1.01	(1.00 - 1.03	0.0906	1.35	(0.85 - 2.14	0.1984

Of the 20 genes in cluster 1, 17 were tested by qPCR (three collagen alpha variants were excluded; *COL1A2, COL5A2, COL6A3*). Furthermore *PAPPA*, which was in cluster 1 and was tested by qPCR, was excluded during quality control. All 17 genes from cluster 1 that were tested by qPCR were strongly associated with ECA (P<0.0036). Of the genes that were selected for qPCR testing based on prior literature (total = 29), 10 were associated with ECA. These included pro-inflammatory chemokines and cytokines *CCL2, CCL3, CXCL5*, *IL1B* and *IL6*, matrix metalloproteinases *MMP1* and *MMP9, CHUK*, *FUT4* and *PTPRC*.

In order to determine whether any of the associations between gene expression and ECA were modulated by doxycycline, regression models were run again using data from baseline and one month only (since treatment was only administered for 28 days after surgery). There was no strong evidence that any models including the interaction fitted the data better than the models without the interaction term ( Table 7), suggesting that doxycycline treatment did not modulate the association between conjunctival gene expression and ECA. There was weak evidence for interactions in *CD68*, *IL10, IFNG* and *MMP12*.

**Table 7.** Evidence for interaction between doxycycline treatment and gene expression in their effect on ECA. P values are shown for each gene of a likelihood ratio test, examining whether a mixed effects logistic regression model including an interaction term between treatment arm and gene expression explained the data better than a model without the interaction term. P values <0.001 are considered significant after adjustment for the 5% FDR.

Target	lrtest P value for interaction
AEBP1	0.1801
CCL2	0.7085
CCL3	0.5269
CCL4	0.3493
CD247	0.2538
CD68	0.0484
CHUK	0.6818
COL1A1	0.8079
COL3A1	0.9109
COL5A1	0.3802
COL6A1	0.6208
COL7A1	0.4736
CSF1R	0.4992
CTGF	0.3885
CXCL5	0.5454
CXCL8	0.7634
FERMT2	0.6096
FKBP10	0.4435
FN1	0.4894
FUT4	0.0902
IFNG	0.0442
IKBKB	0.3031
IL10	0.0582
IL17A	0.093
IL18	0.5483
IL1B	0.8394
IL6	0.9052
MMP1	0.593
MMP12	0.0262
MMP7	0.3676
MMP9	0.7202
MS4A1	0.4666
NCAM1	0.5781
NFKB1	0.5854
PRRX1	0.869
PTPRC	0.8462
S100A7	0.9637
SPARC	0.5113
SPARCL1	0.8013
TGFB1I1	0.2657
TGFBI	0.4808
TNF	0.1645
TPM2	0.8876
VCAN	0.9594

## Discussion

Here we present evidence that a number of pro-fibrotic and pro-inflammatory genes were upregulated in the conjunctivae of patients who developed ECA following trichiasis surgery. Analysis of 3’RNA sequencing data revealed that all treatment/outcome groups upregulated wound healing pathways at one-month post-surgery, however surprisingly, there were no differences in the expression of individual genes between groups. Visualisation of global gene expression by PCA revealed considerable variation amongst one-month samples relative to baseline samples. Clusters of highly coexpressed genes were identified in the raw data, the largest of which was enriched for pro-fibrotic pathways. The summed expression of this cluster of coexpressed genes was upregulated in individuals with a Poor outcome relative to those with a Good outcome in the placebo group. However, this cluster was not upregulated in the doxycycline-treated Poor outcome group. The expression of the genes in this cluster and a number of other immune response genes hypothesised to be associated with ECA and reported to be immunomodulated by doxycycline were evaluated in a larger number of participants by qPCR at baseline, one and six months. The results showed that all genes tested within this pro-fibrotic cluster and a number of the other pro-inflammatory genes were strongly associated with ECA, adjusting for trial arm, age and sex. No strong evidence was found to suggest that doxycycline treatment had an immunomodulatory effect on the association between gene expression and ECA.

The genes strongly associated with ECA included all tested members of cluster one (enriched for collagen biosynthesis/formation, integrin interactions and extracellular matrix organisation pathways), pro-inflammatory cytokines and chemokines ( *CCL2, CCL3, CXCL5*, *IL1B* and *IL6)*, matrix metalloproteinases *MMP1* and *MMP9, CHUK* (also known as IKK-α, an inhibitory regulator of NFκB transcription factor), *FUT4* (also known as CD15, a cellular marker of granulocytes), and *PTPRC* (also known as CD45 or common leukocyte antigen). All associated genes were upregulated in individuals with ECA.

The upregulation of pro-inflammatory mediators and cell markers ( *CCL2, CCL3, CXCL5*, *IL1B, IL6, CHUK, FUT4* and *PTPRC*) is indicative of higher levels of inflammation and leukocyte infiltration in the conjunctival tissue of patients who developed ECA following trichiasis surgery. Inflammation is a crucial stage of the wound healing process, necessary to attract leukocytes to the wound site to remove debris and pathogens and to trigger the subsequent proliferative stage. Excessive inflammation can lead to fibrotic pathology; however an increase in pro-inflammatory cytokines was associated with poor prognosis following glaucoma filtration surgery ^31^. The factors underlying the increased inflammation in these individuals is unclear; there was no difference in baseline clinical inflammation grade between those that did and did not develop ECA. Risk factors associated with ECA development include baseline trichiasis severity and older age ^32, 33^, however there was no difference in baseline age or trichiasis severity in those with and without ECA in this study. Intersurgeon variability and distance between sutures were also associated with ECA development following PLTR surgery ^33^, however there was no difference between surgeon and ECA outcome in this trial.

A number of pro-fibrotic genes were upregulated in individuals with ECA, including *VCAN, FN1, SPARC* and *FERMT2* (proteins that constitute the ECM or regulate the interactions between cells and the ECM), matrix metalloproteinases *MMP1* and *MMP9* and pro-fibrotic growth factors *CTGF, TGFB1* and *TGFB1|1*. MMPs are involved in multiple stages of wound healing and are crucial for matrix remodelling during the tissue regeneration process ^34^. However, excessive production of MMPs is often associated with pathology and overexpression of MMPs 1, 2, 8, 12 and 13 correlated with levels of corneal fibrosis in patients requiring penetrating and deep anterior lamellar keratoplasty ^35^. TGFβ is a key regulator of the wound healing process and regulates cellular differentiation and proliferation, ECM production and wound contraction. TGFβ is also thought to orchestrate fibrotic outcomes following glaucoma filtration surgery ^36^ and it induces tissue contraction and fibronectin production by conjunctival fibroblasts ^37^. Furthermore, increased expression of fibronectin and integrin α5β1 are thought to mediate TGFβ-induced myofibroblast differentiation ^38^, which have high contractile activity. The upregulation of these pro-fibrotic and matrix factors in patients with ECA likely reflects excessive remodelling and contraction of the conjunctival tissue.

Type I, III, V, VI and VII collagen transcripts were strongly upregulated in individuals with ECA and were all found in co-expression cluster one. Collagens are fibrous proteins that make up the connective tissue and collagen type I is the major constituent of the ECM ^39^. Early on during the wound healing process myofibroblasts are thought to produce collagen type III, which is later replaced by collagen type I ^40^. Collagen types III, V and VI have been found to be increased in pathological scarring conditions ^41– 44^. In tissue from normal conjunctiva examined by immunohistochemistry, collagen types I and III were limited to the substantia propria and collagen type V was absent, whereas in inflammatory and scarring trachoma increased collagen type IV was detected in the thickened basement membrane and new collagen type V was deposited in the upper substantia propria ^45, 46^. A number of collagen transcripts and proteins, in particular collagen types I, VIII and XI, were associated with pathological scarring in the conjunctiva following glaucoma filtration surgery in mice and humans ^13^.

Contrary to our hypothesis, we found no strong evidence for modulation of the association between gene expression and ECA development by doxycycline treatment. In support of this finding there was no evidence that doxycycline treatment reduced the risk of developing post-operative trichiasis or ECA in the overall trial ^5^. These findings are somewhat surprising given the evidence supporting the clinical use of oral doxycycline to reduce inflammation and fibrosis in various diseases including rosacea, idiopathic pulmonary fibrosis, chronic blepharitis, corneal erosion and corneal melting post- *Pseudomonas* infection ^19– 21, 47– 49^. Furthermore, doxycycline treatment *in vitro* reduced MMP1, 9 and 12 expression by primary fibroblasts from trichiasis patients and reduced collagen matrix contraction ^22^. Treatment was taken every day for 28 days prior to collection of the one-month sample therefore levels of active drug in the body should have remained high, and the positive impact of oral doxycycline on corneal and other skin diseases suggests that it successfully penetrates the epithelium. Oral doxycycline administered for three weeks was found to have no benefit in patients with persistent symptoms following treatment for neuroborreliosis (a neurological manifestation of tick-borne Lyme disease); there was no difference in symptoms relative to a placebo group and no change in systemic cytokine responses ^50^. It is possible that in the context of trichiasis surgery, doxycycline either has no immunomodulatory effect or it does not immunomodulate the factors involved in recurrent trichiasis and ECA.

Although a large number of genes were upregulated in all treatment/outcome groups at one-month post-surgery by RNA sequencing, no differences were detected between groups. This was probably due to the small sample size and high degree of variation in the one-month samples, evident by PCA. Reducing this variation through summed expression of coexpressed gene clusters revealed differences in wound healing pathways that were validated by qPCR. A strength of this study was the original trial design, including standardisation of surgery, patient treatment adherence and follow up. This study was designed and targets were chosen for follow-up to investigate the immuno-fibrogenic factors associated with the development of ECA, therefore it was not appropriate to reanalyse the results in relation to trachomatous trichiasis recurrence (reported elsewhere ^10^) or ECA regression; in addition, these analyses would have had small group sizes.

This study has revealed that the development of post-operative ECA is strongly associated with the upregulation of pro-fibrotic genes, including growth factors (and the canonical fibrogenic growth factor *TGFB1*), matrix proteins and collagens. A number of MMPs and pro-inflammatory cytokines were also upregulated. Contrary to our hypothesis, there was no strong evidence to suggest that doxycycline modulated the relationship between gene expression and ECA. Although it is reassuring that ECA regressed in roughly a third of patients by 6 months, the short-term aesthetic appearance may still dissuade others from undergoing trichiasis surgery and anti-fibrotic treatments to prevent ECA development remain desirable. 

## Data availability

### Underlying data

Raw and processed RNAseq data for ‘The conjunctival transcriptome in Ethiopians after trichiasis surgery: associations with the development of eyelid contour abnormalities and the effect of oral doxycycline treatment’, Accession number GSE135455: https://identifiers.org/geo:GSE135455

Figshare: The conjunctival transcriptome in Ethiopians after trichiasis surgery: associations with the development of eyelid contour abnormalities and the effect of oral doxycycline treatment, https://doi.org/10.6084/m9.figshare.9696143.v1 ^51^

This project contains the following underlying data:

- Raw and processed qPCR data

Data are available under the terms of the Creative Commons Attribution 4.0 International license (CC-BY 4.0).

### Extended data

Figshare: EXTENDED DATA [The conjunctival transcriptome in Ethiopians after trichiasis surgery: associations with the development of eyelid contour abnormalities and the effect of oral doxycycline treatment], https://doi.org/10.6084/m9.figshare.9696263.v1 ^52^

This project contains the following extended data:

- Clusters of co-expressed genes in the raw read count matrix for all samples. Using a Pearson correlation cut-off of 0.88, 40 clusters were identified. Panel A shows the names of genes in each cluster. Panel B shows the ten most enriched pathways for the genes in each cluster. Panel C shows results of differential expression testing of the cluster-sum (summed read counts of all genes in the cluster for each individual), adjusting for baseline cluster-sum expression, age and sex. Each group was compared to the reference group of Placebo-Good outcome.

Data are available under the terms of the Creative Commons Zero "No rights reserved" data waiver (CC0 1.0 Public domain dedication).
