# Supplementary material for: The conjunctival transcriptome in Ethiopians after trichiasis surgery: associations with the development of eyelid contour abnormalities and the effect of oral doxycycline treatment
Source: Wellcome Open Res. 2022 Nov 11;4:130. Originally published 2019 Sep 4. [Version 2] doi: 10.12688/wellcomeopenres.15419.2 (PMC10323279; doi:10.12688/wellcomeopenres.15419.2)
Supplement: Peer review history

# Peer review: Reviewer response for version 2

**DOI:** 10.21956/wellcomeopenres.20379.r61390

**Published:** 2023-07-28

**Authors:** Ka-Wai Kam

**Affiliations:** 1 Department of Ophthalmology and Visual Sciences, Prince of Wales Hospital, The Chinese University of Hong Kong, Hong Kong, Hong Kong

The authors conducted a beautiful study and the manuscript was well written.

 Regarding the demographics, subjects with poor outcome were significantly older. Was there any interaction between age and genetics underlying the pathogenesis of ECA?

 This could have been published elsewhere (in the methodology paper), but how did the investigator measure compliance of subjects to doxycycline treatment?

Is the work clearly and accurately presented and does it cite the current literature?

Yes

If applicable, is the statistical analysis and its interpretation appropriate?

Yes

Are all the source data underlying the results available to ensure full reproducibility?

Yes

Is the study design appropriate and is the work technically sound?

Yes

Are the conclusions drawn adequately supported by the results?

Yes

Are sufficient details of methods and analysis provided to allow replication by others?

Yes

Reviewer Expertise:

Epidemiology

I confirm that I have read this submission and believe that I have an appropriate level of expertise to confirm that it is of an acceptable scientific standard.

# Peer review: Reviewer response for version 2

**DOI:** 10.21956/wellcomeopenres.20379.r61396

**Published:** 2023-07-05

**Authors:** Swati Singh

**Affiliations:** 1 Ophthalmic Plastic Surgery, Orbit and Ocular Oncology Services, Centre for Ocular Regeneration, L V Prasad Eye Institute, Hyderabad, Telangana, India

The authors have analyzed the transcriptional changes associated with the early development of ECA and the impact of doxycycline. The study design has looked at pro- and antifibrotic pathways, which is very interesting. It is a new approach to addressing ECA's occurrence post-trichiasis surgery and worth publishing. Few comments are:

1. The methodology is extensive, and authors could add a flow chart depicting their steps-esp. Sample processing. 
2. Conjunctival swabs represent the gene expression of tears and superficial cells of the tarsal conjunctiva. Fibrosis or ECA would have more changes in subconjunctival tissue; hence one month of follow-up, which is anyways period of tissue healing (varies in each person), could give insignificant results. 

Is the work clearly and accurately presented and does it cite the current literature?

Yes

If applicable, is the statistical analysis and its interpretation appropriate?

Yes

Are all the source data underlying the results available to ensure full reproducibility?

Yes

Is the study design appropriate and is the work technically sound?

Yes

Are the conclusions drawn adequately supported by the results?

Yes

Are sufficient details of methods and analysis provided to allow replication by others?

Yes

Reviewer Expertise:

Dry eye disease, ocular surface, eyelids, eyelashes

I confirm that I have read this submission and believe that I have an appropriate level of expertise to confirm that it is of an acceptable scientific standard.

# Peer review: Reviewer response for version 1

**DOI:** 10.21956/wellcomeopenres.16862.r41831

**Published:** 2021-02-01

**Authors:** Hon Shing Ong

**Affiliations:** 1 Corneal and External Diseases Department, Singapore National Eye Centre, Singapore, Singapore; 2 Tissue Engineering and Cell Therapy Group, Singapore Eye Research Institute, Singapore, Singapore

This study sought to investigate the transcriptional changes associated with the early development of eyelid contour abnormalities (ECA) following trichiasis surgery. It also aimed to evaluate the effects of doxycycline on such transcription patterns.

 The research question is important. Despite being a significant cause of ocular morbidity, the underlying mechanisms of ECA development following trichiasis surgery is unclear. This translates to a lack of effective therapies for the prevention or treatment of ECA.  ECA is also an important reason why patients with trachoma refuse sight-saving trichiasis surgeries. Thus, understanding the transcriptional changes is an important step forward in figuring out the pathophysiology of ECA and the development of potentially more effective therapies. Furthermore, doxycycline is a widely used drug for inflammatory disorders of the ocular surface. Establishing the anti-inflammatory and anti-fibrotic effects of oral doxycycline in trachomatous lid pathologies is thus clinically relevant.

 The study was designed within a randomised, double-masked, placebo-controlled trial. This is appropriate to address the study hypothesis. The work performed by the authors has been presented well with methodologies and analyses clearly described.

 Some minor points to consider:

1. Abstract: ‘12 in each treatment/ECA outcome group’ requires more description. From the abstract, it is unclear to the reader, what these groups are. It is only available in the methods section of the main manuscript.
2. Methods: The nurse-surgeons have been described as ‘experienced’. Perhaps a quantification of the number of years of experience (e.g. median or range) of the nurses should be included. Also, how many nurse-surgeons were involved in this study?
3. Methods: As this is within an RCT, the method of randomisation should be described.
4. Methods: Were there specific exclusion criteria (e.g. patients already on specific anti-inflammatory drugs)?
5. Methods: Of the 98 samples that failed QC, which groups were the samples from? Was the number of failed samples relatively equal in the moderate/severe ECA group compared to samples taken from individuals without/mild ECA?
6. Methods: Details of the power calculation for both analyses should be included. Were the sample sizes adequate to detect differences between the groups for the RNA seq experiments? Also, the power of the study is relevant as the study reported results showing no evidence of doxycycline treatment affecting gene expression and ECA development.
7. Results: The first paragraph of the results have been described in the Methods section and can probably be left out.
8. Results: For table 1, were there significant differences in the baseline characteristics between the groups?
9. Results: For table 2, it appears that more genes were up-regulated in poor outcome groups compared to control groups. Also, there were a higher number of differentially expressed genes in the ‘doxycycline-good’ group compared to ‘placebo-good’ group. Were these statistically significant?
10. Results: For table 5, were there significant differences in the baseline clinical and demographic characteristics between the groups?
11. Discussion: The authors could perhaps include a short paragraph on the relevance of their transcriptome findings on understanding the pathophysiology of ECA development and how this is important in the future development of targeted anti-inflammatory or anti-fibrotic therapies.

Is the work clearly and accurately presented and does it cite the current literature?

Yes

If applicable, is the statistical analysis and its interpretation appropriate?

Partly

Are all the source data underlying the results available to ensure full reproducibility?

Yes

Is the study design appropriate and is the work technically sound?

Yes

Are the conclusions drawn adequately supported by the results?

Yes

Are sufficient details of methods and analysis provided to allow replication by others?

Yes

Reviewer Expertise:

Various aspects of corneal and external eye diseases, corneal transplantation (Clinical and Translational);Ocular surface inflammation and scarring;Phenotype characterisation and biomarkers of ocular surface scarring diseases (cicatrising conjunctivitis);Cell-based therapies for corneal diseases.

I confirm that I have read this submission and believe that I have an appropriate level of expertise to confirm that it is of an acceptable scientific standard.

# Response

**Published:** 2022-09-07

**Authors:** Tamsyn Derrick

**Affiliations:** London School of Hygiene & Tropical Medicine, UK

Abstract: ‘12 in each treatment/ECA outcome group’ requires more description. From the abstract, it is unclear to the reader, what these groups are. It is only available in the methods section of the main manuscript.

**This has been clarified**

 Methods: The nurse-surgeons have been described as ‘experienced’. Perhaps a quantification of the number of years of experience (e.g. median or range) of the nurses should be included. Also, how many nurse-surgeons were involved in this study?

**We have added additional information of the prior experience of surgeons to lines 139-142**

 Methods: As this is within an RCT, the method of randomisation should be described.

**Full trial details are published elsewhere and this statement has been added to the methods.**

 Methods: Were there specific exclusion criteria (e.g. patients already on specific anti-inflammatory drugs)? 

**As above, full trial details including exclusion criteria are published elsewhere and this statement has been added to the methods.**

 Methods: Of the 98 samples that failed QC, which groups were the samples from? Was the number of failed samples relatively equal in the moderate/severe ECA group compared to samples taken from individuals without/mild ECA?

**As described above, the samples that failed did not differ from those that passed QC by age, sex, or group. A sentence has been added to clarify this and the numbers in each group are shown to reviewer 1**

 Methods: Details of the power calculation for both analyses should be included. Were the sample sizes adequate to detect differences between the groups for the RNA seq experiments? Also, the power of the study is relevant as the study reported results showing no evidence of doxycycline treatment affecting gene expression and ECA development.

**RNAseq experiments were conceived to be hypothesis generating for TLDA target selection. The number of samples selected was limited by RNA quality and sufficiency for RNAseq and the total budget available. The a priori design of this nested study within a clinical trial, was the assessment of gene expression profiles in all people developing ECA (and a matched control group).  As the numbers that would go on to develop ECA were not known beforehand a separate sample size calculation for this outcome was not performed and a post-hoc power calculation is not a recommended practice.”**

 The first paragraph of the results have been described in the Methods section and can probably be left out.

**This has been removed**

 For table 1, were there significant differences in the baseline characteristics between the groups?

**Samples were selected for RNA-seq based upon trachomatous scarring and eyelid distortion characteristics at baseline, one and six months, as described on line 183. There was no difference in sex between groups (Chi2 P value 0.763), however age distribution differed slightly between groups (ANOVA P value 0.0143).**

 For table 2, it appears that more genes were up-regulated in poor outcome groups compared to control groups. Also, there were a higher number of differentially expressed genes in the ‘doxycycline-good’ group compared to ‘placebo-good’ group. Were these statistically significant

**This observation is described in the first paragraph of the results section; As this was a descriptive testing for differences between total numbers of differentially expressed genes was not performed.**

 For table 5, were there significant differences in the baseline clinical and demographic characteristics between the groups?

**Groups with no or mild ECA at one month were matched to those with moderate/severe ECA by age, sex and trial arm. There was no difference between groups in baseline conjunctival scarring (Chi2 P value 0.192), age (ANOVA P value 0.0737) or sex (Chi2 P value 0.162). *C. trachomatis* was detected in only 8 samples therefore differences between groups were not tested.**

 The authors could perhaps include a short paragraph on the relevance of their transcriptome findings on understanding the pathophysiology of ECA development and how this is important in the future development of targeted anti-inflammatory or anti-fibrotic therapies.

**The final paragraph of the discussion has been updated to address this point.**

# Peer review: Reviewer response for version 1

**DOI:** 10.21956/wellcomeopenres.16862.r36397

**Published:** 2019-10-04

**Authors:** Emily W. Gower

**Affiliations:** 1 Department of Epidemiology, University of North Carolina at Chapel Hill, Chapel Hill, NC, USA

This manuscript reports data from a novel study investigating the association between gene expression patterns and eyelid contour abnormality development following trichiasis surgery. This is an important study, as little is known about the pathophysiology of ECAs in this population. 

 The manuscript is well written and describes in detail the analyses used.

 Prior research has demonstrated that the immediate post-operative appearance has a significant impact on both return of trichiasis and the development of eyelid contour abnormalities. At present, the manuscript does not address this issue. The following recommendations should be considered for addressing this gap: 

- In the introduction, paragraph 3, it would be useful to discuss the existing literature on the association between surgical appearance and subsequent ECAs (where the authors discuss that the pathophysiology is unknown).
- In a masked fashion, review the post-operative photos (either immediate or 1 day) to assess the eyelid contour for the 48 individuals included in the primary analyses and report whether there are differences between groups. 
- Evaluate the impact of the post-operative contour on the genetic results. It is likely that both the contour and the underlying genetic upregulation both impact the development of ECA, and possibly the regression of ECAs seen in the early post-operative period, but not at later visits. 

Methods:

 Exam section, last paragraph: Please clarify that it is the conjunctival scarring that was graded with the FPC system.

 Last paragraph before data analysis: Were the samples that failed randomly distributed or were they more commonly from one or more groups - either one of the 4 groups, or did they vary by age and/or sex.

 There is a bit of a mix of methods in the results and results in the methods section. Recognizing that sometimes it is important to describe results of intermediate steps in the methods when they influence the next set of methods, please review and consider not repeating specific aspects. For example, the first paragraph of the results is nearly a copy/paste from the methods.

 Discussion:

 Bottom of page 12: Please include discussion regarding prior research that has shown a strong association between immediate post-op appearance and early outcomes. [Merbs, PLOS NTD 2012 reports that 85% of the time ECAs were predicted based on immediate post-op appearance]

 Minor suggestions: 

 In the introduction the word “recurrence” is used instead of post-operative trichiasis. In keeping with WHO recommendation, it would be good to use the latter instead.

 The introduction reports 12-22% “recurrence” within 12 months after surgery and then cites the study used for this analysis and one other by this group. There is a much broader range of PTT rates, and studies have been reported by other groups. This statement should be modified to reflect the range of rates reported in the literature.

 Please check references in the 3 ^rd^ paragraph of the introduction. At present, the second sentence references the grading scheme for ECAs, but is discussing the corneal damage and vision loss associated with ECAs. 

 Same paragraph, “patient’s” should be “patients’”

 Second to last paragraph of introduction: please add numbers or %s regarding ECAs, as was done with the TT numbers.

Is the work clearly and accurately presented and does it cite the current literature?

Partly

If applicable, is the statistical analysis and its interpretation appropriate?

Yes

Are all the source data underlying the results available to ensure full reproducibility?

Yes

Is the study design appropriate and is the work technically sound?

Yes

Are the conclusions drawn adequately supported by the results?

Partly

Are sufficient details of methods and analysis provided to allow replication by others?

Yes

Reviewer Expertise:

Epidemiology, trichiasis surgery Note: This reviewer does not have a strong background in genetic analysis. As such, it would be useful for someone with that skillset to review the genetic methods.

I confirm that I have read this submission and believe that I have an appropriate level of expertise to confirm that it is of an acceptable scientific standard, however I have significant reservations, as outlined above.

# Response

**Published:** 2022-09-07

**Authors:** Tamsyn Derrick

**Affiliations:** London School of Hygiene & Tropical Medicine, UK

**The introduction has been updated as suggested.**

**We agree that it would be interesting to look at post-operative appearance and ECA development in this group and at the transcriptomic level. This study was not designed to look at this question and we think that it is not in scope for this paper. We did not take photos immediately after surgery or day-one postop. This gap has now been highlighted in the discussion as potentially requiring further work.**

**We have clarified that the FPC system was used in grading**

**We have added that the samples that failed QC did not differ from those that passed QC by age, sex, or group. A sentence has been added on lines 247-250 to clarify this and the numbers in each group are shown below.**

**Doxy_good**

**Doxy_poor**

**Placebo_good**

**Placebo_poor**

**Excluded samples**

**37 (39.4%)**

**7 (7.5%)**

**40 (42.6%)**

**10 (10.6%)**

**Included samples**

**300 (38.8%)**

**92 (11.9%)**

**295 (38.1%)**

**87 (11.2%)**

**We removed the repeated paragraph from the results.**

**We added the suggested Merbs et al citation to the discussion**

**All instances of ‘recurrence’ in the paper have been changed to ‘post-operative trichiasis’.**

**We agree there is a wider range of reported outcomes. This has been adjusted to reflect this. We believe the most suitable references for this is the review conducted by Rajak et al ( Trachomatous Trichiasis and its management in endemic countries) and Cochrane review  by Burton et al ( Interventions for trachomatous trichiasis). We have revised the sentence and added these two references.**

**We have removed the incorrect citation in the introduction and corrected patients**

**We have added figures  regarding ECAs as requested.**
